# Angiotensin-(1-7) attenuates disuse skeletal muscle atrophy in mice via its receptor, Mas

**DOI:** 10.1242/dmm.023390

**Published:** 2016-04-01

**Authors:** María Gabriela Morales, Johanna Abrigo, María José Acuña, Robson A. Santos, Michael Bader, Enrique Brandan, Felipe Simon, Hugo Olguin, Daniel Cabrera, Claudio Cabello-Verrugio

**Affiliations:** 1 Laboratory of Biology and Molecular Physiopathology, Department of Biological Sciences, Faculty of Biological Sciences & Faculty of Medicine, Universidad Andrés Bello, Santiago 8370146, Chile; 2 Millennium Institute on Immunology and Immunotherapy, Santiago 8370146, Chile; 3 Center for Cell Regulation and Pathology (CRCP), Center for Regeneration and Aging (CARE), Laboratory of Cell Differentiation and Pathology, Department of Cell and Molecular Biology, Faculty of Biological Sciences, P. Universidad Católica de Chile, Santiago 8331150, Chile; 4 National Institute in Science and Technology in Nanobiopharmaceutics, Department of Physiology and Biophysics, Federal University of Minas Gerais (UFMG), Belo Horizonte 31270-901, Brazil; 5 Max-Delbrück-Center for Molecular Medicine, Berlin-Buch 13125, Germany; 6 National Institute in Science and Technology in Nanobiopharmaceutics, Belo Horizonte 31270-901, Brazil; 7 Laboratory of Integrative Physiopathology, Department of Biological Sciences, Faculty of Biological Sciences & Faculty of Medicine, Universidad Andrés Bello, Santiago 8370146, Chile; 8 Laboratory of Tissue Repair and Adult Stem Cells, Department of Cell and Molecular Biology, Faculty of Biological Sciences, P. Universidad Católica de Chile, Santiago 8331150, Chile; 9 Departamento de Ciencias Químicas y Biológicas, Facultad de Salud, Universidad Bernardo O Higgins, Santiago 8370993, Chile; 10 Departamento de Gastroenterología, Facultad de Medicina, Pontificia Universidad Católica de Chile, Santiago 8330024, Chile

**Keywords:** Disuse, Angiotensin-(1-7), Mas receptor, Skeletal muscle, Atrophy

## Abstract

Immobilization is a form of disuse characterized by a loss of strength and muscle mass. Among the main features are decreased IGF-1/Akt signalling and increased ubiquitin-proteasome pathway signalling, which induce greater myosin heavy chain degradation. Activation of the classical renin-angiotensin system (RAS) causes deleterious effects in skeletal muscle, including muscle wasting. In contrast, angiotensin-(1-7) [Ang-(1-7)], a peptide of the non-classical RAS, produces beneficial effects in skeletal muscle. However, the role of Ang-(1-7) in skeletal muscle disuse atrophy and independent of classical RAS activation has not been evaluated. Therefore, we assessed the functions of Ang-(1-7) and the Mas receptor in disuse muscle atrophy *in vivo* using unilateral cast immobilization of the hind limb in male, 12-week-old wild-type (WT) and Mas-knockout (Mas KO) mice for 1 and 14 days. Additionally, we evaluated the participation of IGF-1/IGFR-1/Akt signalling and ubiquitin-proteasome pathway expression on the effects of Ang-(1-7) immobilization-induced muscle atrophy. Our results found that Ang-(1-7) prevented decreased muscle strength and reduced myofiber diameter, myosin heavy chain levels, and the induction of atrogin-1 and MuRF-1 expressions, all of which normally occur during immobilization. Analyses indicated that Ang-(1-7) increases IGF-1/IGFR-1/Akt pathway signalling through IGFR-1 and Akt phosphorylation, and the concomitant activation of two downstream targets of Akt, p70S6K and FoxO3. These anti-atrophic effects of Ang-(1-7) were not observed in Mas KO mice, indicating crucial participation of the Mas receptor. This report is the first to propose anti-atrophic effects of Ang-(1-7) via the Mas receptor and the participation of the IGF-1/IGFR-1/Akt/p70S6K/FoxO3 mechanism in disuse skeletal muscle atrophy.

## Abstract (precis)

**Summary:** In this article, the authors demonstrate that a peptide with actions mainly in the cardiovascular system prevents the skeletal muscle damage induced by disuse.

## INTRODUCTION

Disuse muscle atrophy is induced by low mechanical load (e.g. cast immobilization) (Bodine, 2013). Morphological changes of disuse include decreased muscle mass, cross-sectional muscle fiber area, and strength (Bodine, 2013). Muscle mass maintenance depends on protein synthesis and degradation equilibrium, which are unbalanced during muscle wasting (Brooks and Myburgh, 2014).

In normal muscle, insulin-like growth factor-1 (IGF-1), via IGFR-1, activates protein kinase B (Akt), resulting in p70S6 kinase (p70S6K) phosphorylation and activation. During muscle atrophy, activity of the IGF-1/Akt protein-synthesis pathway is decreased (Latres et al., 2005; Clemmons, 2009; Frost and Lang, 2007). Protein degradation increases during muscle wasting, primarily affecting the skeletal muscle proteins myosin heavy chain (MHC) and actin (Diffee et al., 2002; Meneses et al., 2015). Regarding this, the ubiquitin-proteasome pathway (UPP) mediates most myofibrillar protein degradation in skeletal muscle atrophy (Lecker et al., 1999). The atrophy F-box protein (MAFbx; also known as atrogin-1) and RING-finger protein-1 (MuRF-1), muscle-specific E3 ligases, are upregulated in various skeletal muscle wasting models (Foletta et al., 2011) and are gene targets of Forkhead box, class O (FoxO) transcription factors (Sandri, 2008).

The renin-angiotensin system (RAS), which comprises classical and non-classical axes, is a regulator of muscle mass (Cabello-Verrugio et al., 2012a). The main classical RAS peptide is angiotensin II (Ang II), which is associated with deleterious effects in skeletal muscle (Cabello-Verrugio et al., 2015). Angiotensin-(1-7) [Ang-(1-7)], the main non-classical RAS peptide, exerts actions against Ang II via the G-protein-coupled receptor Mas (Santos et al., 2003). These contrasting functions prevent the insulin resistance, fibrosis, and autonomic dysfunction associated with Duchenne muscular dystrophy and Ang-II-induced skeletal muscle atrophy (Acuna et al., 2014; Cabello-Verrugio et al., 2015; Riquelme et al., 2014). Ang-(1-7) minimizes Ang-II-induced muscle wasting through UPP reduction by decreasing atrogin-1 and *MuRF-1* mRNA expressions, as well as by maintaining MHC levels. Moreover, Ang-(1-7), via the Mas receptor, induces Akt phosphorylation in skeletal muscle (Cisternas et al., 2015).

However, the role of Ang-(1-7), as a mechanism independent of classical RAS activation, has not been evaluated in skeletal muscle disuse atrophy. Therefore, the present study evaluated the effects of Ang-(1-7) and the Mas receptor using a unilateral cast immobilization model in mice (Krawiec et al., 2005; Morales et al., 2015). Systemic Ang-(1-7) treatment prevented the atrophic effects of disuse, restored muscle strength, and inhibited decreased muscle diameter and mass in tibialis anterior (TA) and gastrocnemius (GA) muscles of hind-limb-immobilized mice. All of these effects were blunted in Mas-knockout (Mas KO) mice, suggesting Mas receptor participation in Ang-(1-7) functions. Also through the Mas receptor, Ang-(1-7) decreased atrogin-1 and *MuRF-1* expressions and inhibited the drop in MHC levels normally associated with disuse. Finally, in basal and disuse conditions, Ang-(1-7), through the Mas receptor, increased IGF-1 expression and induced IGFR-1, Akt, p70S6K and FoxO3 phosphorylation, suggesting also the modulation of signalling pathways involved in protein synthesis.

## RESULTS

### Ang-(1-7) prevents the decreased muscle strength of disuse atrophy through the Mas receptor

**Fig. 1.**
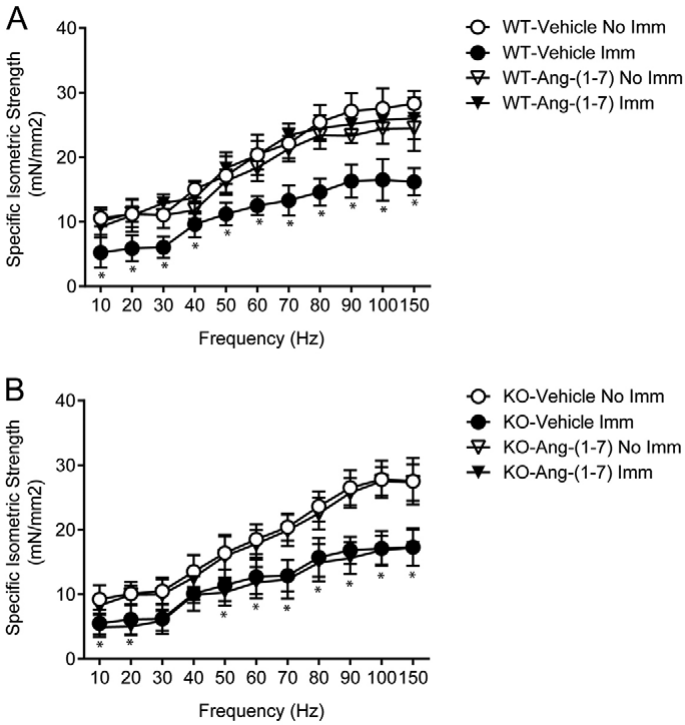
**Angiotensin-(1-7) inhibits the decreased isometric force of muscle under disuse through the receptor Mas.** Tibialis anterior (TA) from (A) wild-type (WT) and (B) Mas knockout (KO) mice treated with vehicle or Ang-(1-7) and unilaterally immobilized (Imm) for 14 days. Maximal isometric strengths (mN/mm^2^) against stimulation frequencies (Hz) were evaluated. Values represent the mean±s.d. of three independent experiments. In each experiment, five to seven mice were used for each experimental condition. *P<0.05 vs vehicle-treated/non-immobilized TA.

Decreased muscle strength is a main feature of cast-immobilization skeletal muscle atrophy. After 14 days, the isometric strength of the vehicle-treated immobilized TA muscle in wild-type (WT) mice (WT/immobilized TA) was less than that of WT/non-immobilized TA for all evaluated points on the frequency-strength curve (Fig. 1A). Ang-(1-7) pre-treatment prevented this decrease in isometric strength, with WT/immobilized TA showing values similar to WT/non-immobilized TA. Interestingly, Ang-(1-7)- and vehicle-treated WT/non-immobilized TA showed the same muscle strength (Fig. 1A).

To evaluate Mas receptor participation in minimizing the effects of disuse atrophy, the unilateral immobilization protocol was also implemented in the hind limb of Mas KO mice. Isometric strength in Mas KO/immobilized TA treated with a vehicle was similar to that of WT/immobilized TA treated with a vehicle (Fig. 1A,B). In contrast to WT mice, Ang-(1-7) pre-treatment did not improve isometric strength in TA-immobilized Mas KO mice (Fig. 1B).

Maximal isometric force was also determined. Ang-(1-7) pre-treatment prevented a decrease in maximal isometric force for WT/immobilized TA (Fig. S1A). In contrast, Ang-(1-7) pre-treatment did not prevent this decrease in Mas KO/immobilized TA (Fig. S1B). Notably, the maximal isometric strengths of vehicle-treated WT/− (WT/non-immobilized TA) and Mas KO/non-immobilized TA were similar. In addition, the strength decreased in the same proportion in WT/− and Mas KO/immobilized TA (35% and 38%, respectively).

These effects on isometric strength were also supported by evaluations of the GA muscle (Fig. S1C,D). Specifically, the immobilization-induced decrease in the tetanic isometric force of the GA was prevented by Ang-(1-7) pre-treatment via the Mas receptor.

These results indicate that Ang-(1-7), through the Mas receptor, prevents the disuse loss of muscle strength in the TA and GA.

### Ang-(1-7) inhibits the fiber diameter and muscle mass reductions of disuse atrophy through the Mas receptor

**Fig. 2.**
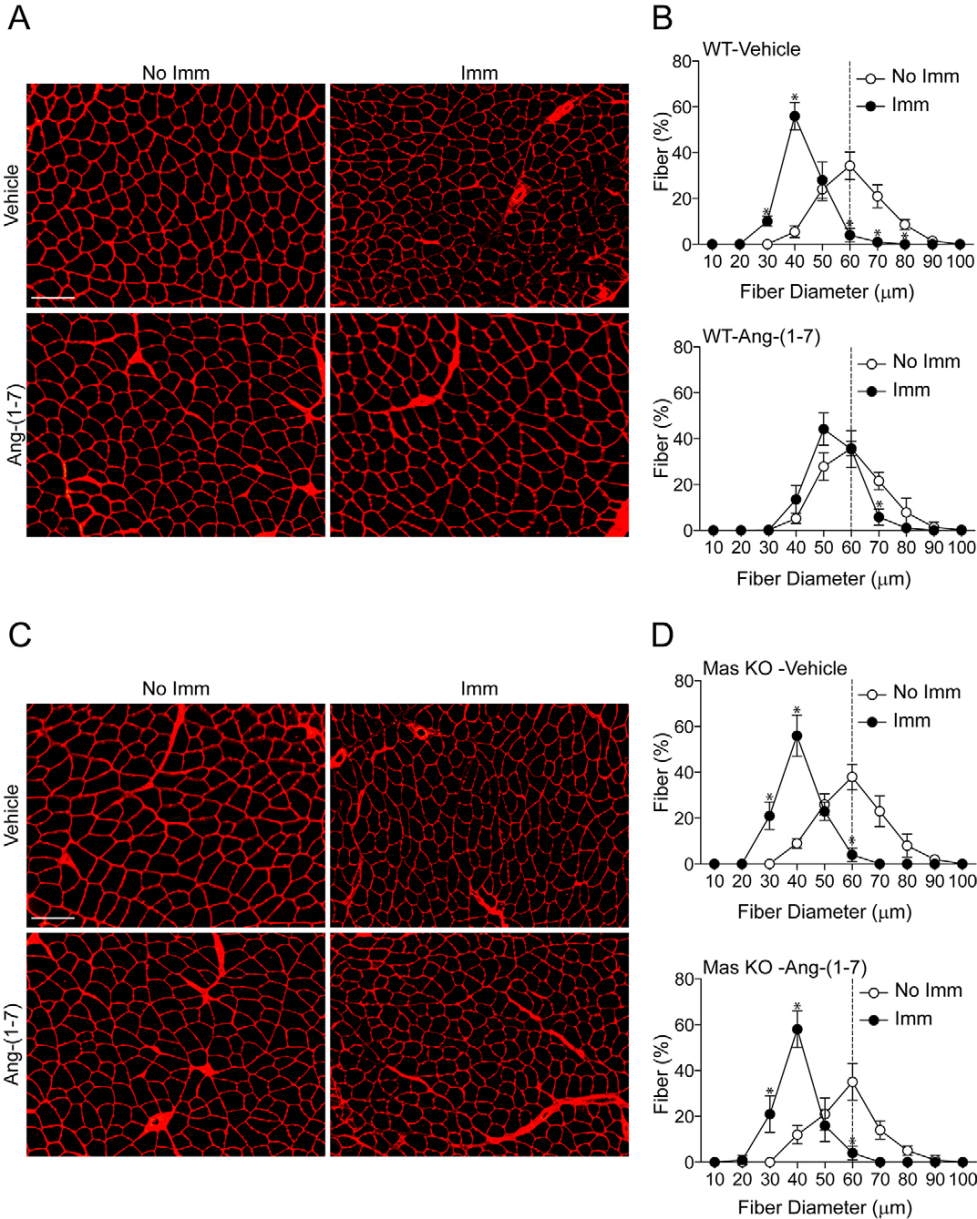
**Angiotensin-(1-7) prevents the decreased fiber diameter of muscle under disuse through the Mas receptor.** The fiber diameter of tibialis anterior (TA) cross-sections from (A,B) wild-type (WT) and (C,D) Mas knockout (KO) mice treated with the vehicle or Ang-(1-7) and unilaterally immobilized for 14 days were compared. (A,C) Muscle cross-sections were stained with WGA to delimit muscle fiber sarcolemma. Scale bars: 150 µm. (B,D) Minimal Feret diameters were determined in TA cross-sections from A and C. Fiber diameters were grouped from 0 to 100 µm. The values are expressed as the percentage of the total quantified fibers, and the image counts are representative of three independent experiments. In each experiment, five to seven mice were used for each experimental condition. Values correspond to the mean±s.d. The dotted line represents the median distribution of minimal Feret diameters observed in vehicle-treated/non-immobilized TA. For better visualization of the changes, the upper panel of B and D compare the diameter of vehicle-treated/non-immobilized TA (No Imm) and vehicle-treated/immobilized TA (Imm), whereas the lower panel shows the same comparison in the Ang-(1-7)-treated group. *P<0.05 vs vehicle-treated/non-immobilized TA.

The effects of Ang-(1-7) on muscle fiber diameters were evaluated (Cisternas et al., 2015; Meneses et al., 2015). The minimal Feret diameters of TA fibers were determined through wheat germ agglutinin (WGA) staining (Fig. 2A), with quantifications showing a shift in the curve towards smaller fibers in vehicle-treated WT/immobilized TA (Fig. 2B, upper graph). This shift was reduced in Ang-(1-7)-treated WT/immobilized TA (Fig. 2B, lower graph), which showed fiber diameters similar to WT/non-immobilized TA. However, Ang-(1-7) did not affect the Mas KO/immobilized TA, which maintained a shift towards smaller fibers similar to vehicle-treated Mas KO/immobilized TA (Fig. 2C,D). These results were also supported by H&E stain in TA (Fig. S2A-D) and in the GA muscle (Fig. S3A-D).

Skeletal muscle atrophy also significantly lowers muscle weight. In this regard, Ang-(1-7) pre-treatment prevented weight loss by 38% in WT/immobilized TA as compared to the vehicle-treated WT/immobilized TA (Fig. S4A). The effect of Ang-(1-7) pre-treatment on muscle weight loss in Mas KO/immobilized TA was null (Fig. S4B). Similar results were obtained in GA muscle from WT (Fig. S4C) and Mas KO mice (Fig. S4D).

These results suggest that Ang-(1-7), through the Mas receptor, decreases the anatomical impacts of disuse atrophy on skeletal muscle.

### Ang-(1-7) inhibits the lowered MHC levels and increased E3 ligase expressions of disuse muscle atrophy through the Mas receptor

**Fig. 3.**
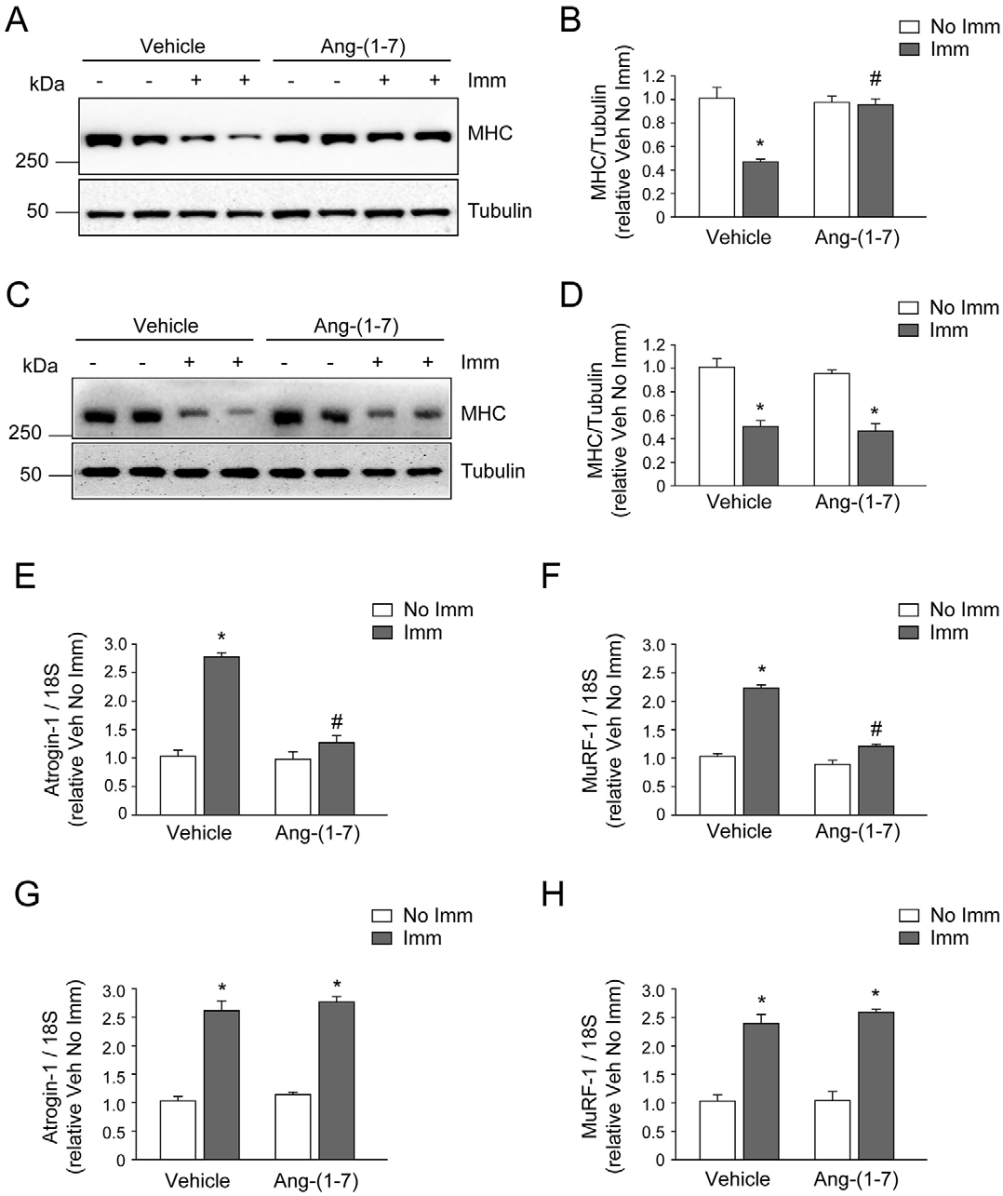
**Angiotensin-(1-7) prevents the decreased myosin heavy chain levels and increased atrogin-1 and *MuRF-1* expressions of muscle under disuse through the Mas receptor.** The hind limb of wild-type (WT) and Mas knockout (KO) mice in the presence or absence of Ang-(1-7) was unilaterally immobilized (Imm) for 14 days. The tibialis anterior (TA) from immobilized (A) WT and (C) Mas KO mice were isolated and homogenized to evaluate myosin heavy chain (MHC) protein levels through western blot analysis. Tubulin levels were used as the loading control. Molecular mass markers are shown in kilodaltons. (B,D) Quantitative analysis of the experiments from A and C, respectively. The levels of MHC normalized to tubulin are expressed relative to vehicle-treated/non-immobilized TA and correspond to the mean±s.d. from three independent experiments. In each experiment, five to seven mice were used for each experimental condition. *P<0.05 vs vehicle-treated/non-immobilized TA; ^#^*P*<0.05 vs vehicle-treated/immobilized TA. Detection of atrogin-1 (E,G) and *MuRF-1* (F,H) mRNA levels through RT-qPCR using 18S as the reference gene. (E,F) WT mice; (G,H) Mas KO mice. Expressions are shown as the fold of induction relative to vehicle-treated/non-immobilized TA and the values correspond to the mean±s.d. of three independent experiments. In each experiment, five to seven mice were used for each experimental condition. *P<0.05 vs vehicle-treated/non-immobilized TA; ^#^*P*<0.05 vs vehicle-treated/immobilized TA.

One of the main proteins affected by skeletal muscle atrophy is MHC, the levels of which decrease through a mechanism involving the UPP. Vehicle-treated WT/immobilized TA presented a 56.5% decline in MHC levels compared to Ang-(1-7)-treated WT/immobilized TA. The MHC levels of Ang-(1-7)-treated WT/immobilized TA were comparable to WT/non-immobilized TA (Fig. 3A,B). In contrast, no Ang-(1-7)-mediated effect was observed in the immobilized TA of Mas KO mice, in which MHC levels decreased by 57.3% and 58.9% in the absence and presence of Ang-(1-7), respectively (Fig. 3C,D).

To further assess UPP participation, the mRNA levels of the ubiquitin E3 ligases atrogin-1 and *MuRF-1* were evaluated. Ang-(1-7) administration respectively prevented antrogin-1 and MuRF-1 inductions by 2.73- and 2.49-fold in WT/immobilized TA as compared to vehicle-treated WT/immobilized TA (Fig. 3E,F). No inhibited atrogin-1 and MuRF-1 upregulation was found in Ang-(1-7)-treated Mas KO/immobilized TA (Fig. 3G,H).

These results indicate that Ang-(1-7) inhibits the lowered MHC levels and increased atrogin-1 and *MuRF-1* expressions that occur during cast immobilization through a mechanism dependent on the Mas receptor.

### Ang-(1-7) activates the IGF-1/IGFR-1/Akt pathway in disuse skeletal muscle atrophy

**Fig. 4.**
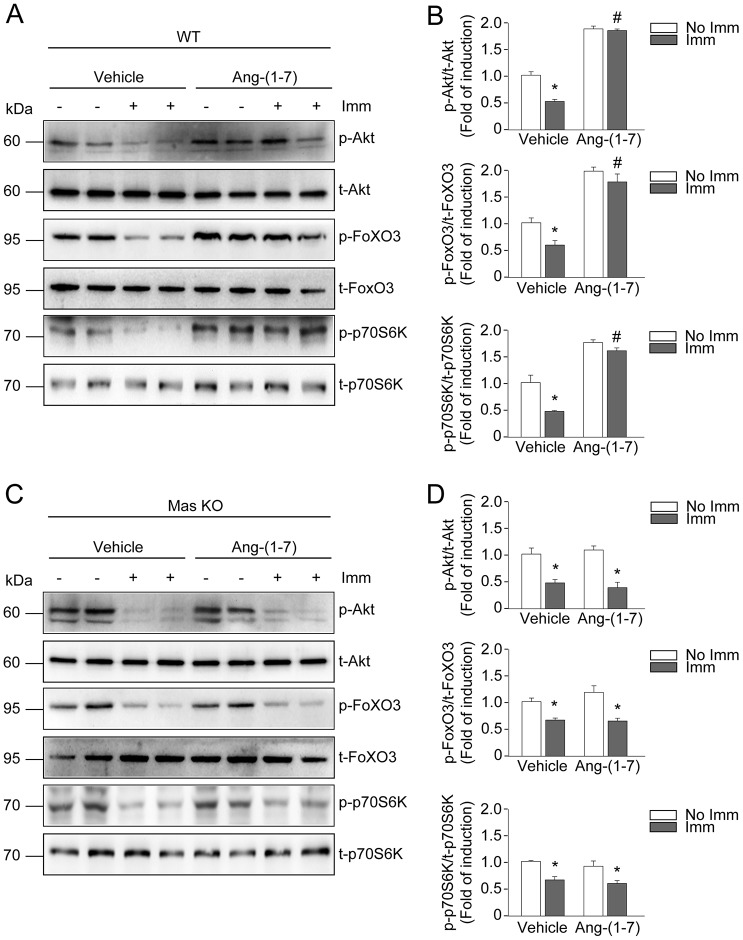
**Angiotensin-(1-7) activates the IGF-1/Akt signalling pathway during muscle disuse through the Mas receptor.** The hind limb of wild-type (WT) and Mas knockout (KO) mice in the presence or absence of Ang-(1-7) were unilaterally immobilized for 14 days. The tibialis anterior (TA) from (A) WT and (C) Mas KO mice were isolated and homogenized to evaluate the protein levels of phosphorylated Akt, FoxO3 and p70S6K via western blot. The total levels of Akt, FoxO3 and p70S6K were used as the loading control. Molecular mass markers are shown in kilodaltons. (B,D) Quantitative analysis of the experiments from A and C, respectively. The levels of phospho-protein normalized to total protein are expressed relative to the vehicle-treated/non-immobilized TA and correspond to the mean±s.d. from three independent experiments. In each experiment, five to seven mice were used for each experimental condition. *P<0.05 vs vehicle-treated/non-immobilized TA; ^#^*P*<0.05 vs vehicle-treated/immobilized TA.

Our group previously showed that Ang-(1-7) abolishes Ang-II-induced skeletal muscle atrophy through a mechanism dependent on Akt activation (Cisternas et al., 2015). However, the actions of Ang-(1-7) on downstream Akt targets in disuse muscle atrophy were unknown. Therefore, Akt phosphorylation was first assessed by western blot. In WT/non-immobilized TA, Ang-(1-7) pre-treatment induced greater Akt phosphorylation as compared to vehicle-treated WT/non-immobilized TA (Fig. 4A). Interestingly, decreased Akt phosphorylation in vehicle-treated WT/immobilized TA was prevented by Ang-(1-7), reaching rates higher than the basal levels evidenced by vehicle-treated WT/non-immobilized TA (Fig. 4A,B).

Next, the phosphorylation of FoxO3, a transcription factor and downstream target of Akt, was evaluated. In the non-immobilized TA of WT hind limb, Ang-(1-7) treatment resulted in higher FoxO3 phosphorylation than did vehicle treatment (Fig. 4A,B). Ang-(1-7) administration also prevented decreased FoxO3 phosphorylation in WT/immobilized TA as compared to the vehicle treatment (Fig. 4A,B).

Then, the activation of p70S6K, another downstream target of Akt, was assessed. p70S6K phosphorylation only slightly decreased in vehicle-treated WT/immobilized TA as compared to WT/non-immobilized TA (Fig. 4A). Similar levels of p70S6K phosphorylation were found in Ang-(1-7)-treated WT/non-immobilized and immobilized TA, which were elevated compared to vehicle-treated WT/non-immobilized TA.

Regarding Ang-(1-7)-treated Mas KO/immobilized TA, Akt, FoxO3 and p70S6K phosphorylation were dependent on the Mas receptor. Thus, Ang-(1-7) was unable to revert the decreased phosphorylation of these targets caused by disuse, whereas phosphorylation levels in the TA of non-immobilized Mas KO mice were similar between groups (Fig. 4C,D).

**Fig. 5.**
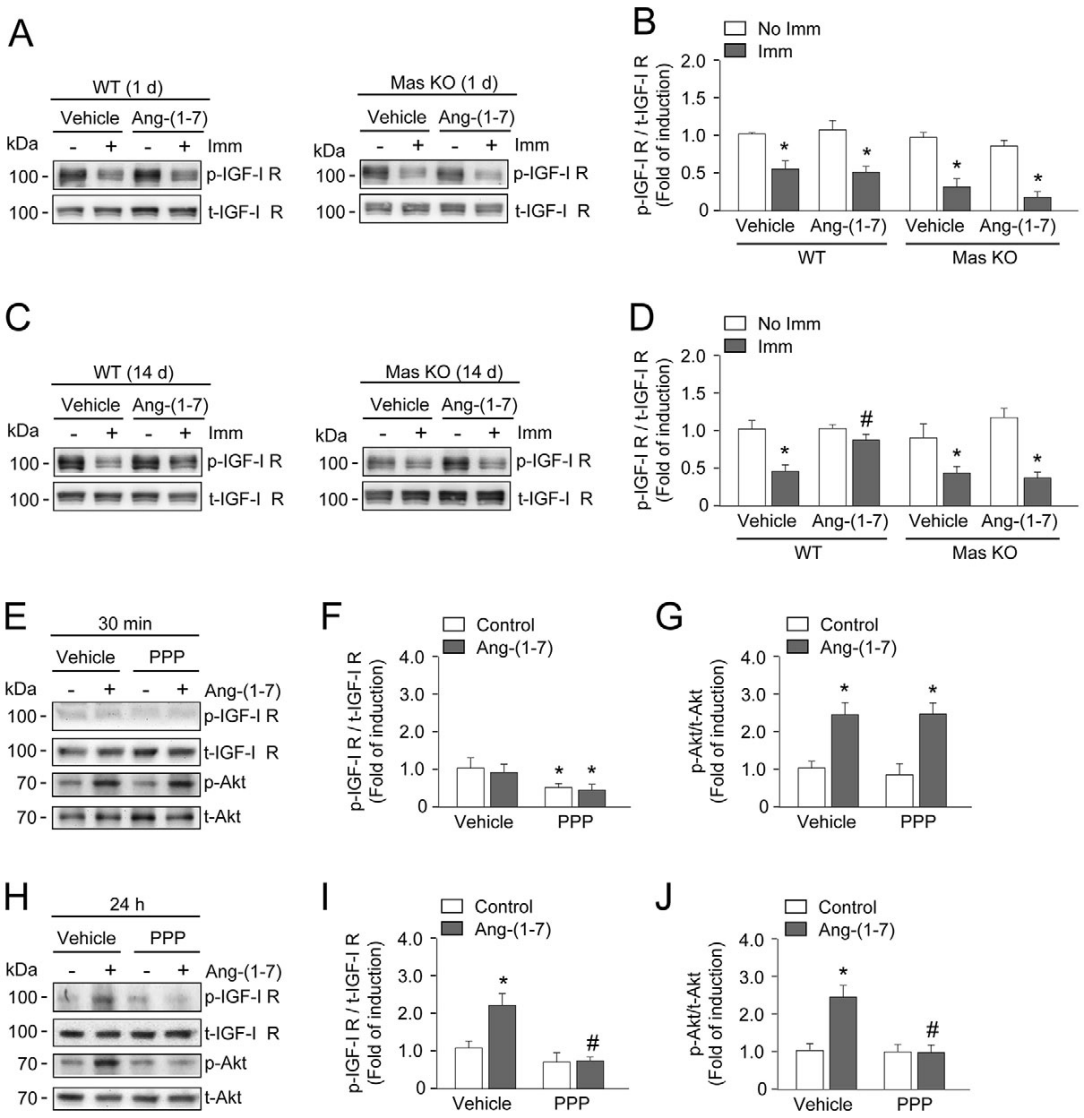
**Angiotensin-(1-7) activates Akt by a mechanism dependent on IGF-1-receptor activation.** Tibialis anterior (TA) from wild-type (WT) and Mas knockout (KO) mice were treated with vehicle or Ang-(1-7) and unilaterally immobilized for 1 (A,B) or 14 (C,D) days. The muscles were homogenized and protein levels of phosphorylated and total IGFR-1 were detected by western blot (A,C). Molecular mass markers are shown in kilodaltons. (B,D) Quantitative analysis of the experiments from A and C, respectively. The levels of phospho-protein normalized to total protein are expressed relative to the vehicle-treated/non-immobilized TA and correspond to the mean±s.d. from three independent experiments. In each experiment, five to seven mice were used for each experimental condition. *P<0.05 vs vehicle-treated/non-immobilized TA; ^#^*P*<0.05 vs vehicle-treated/immobilized TA. (E,H) Primary cultures of myotubes obtained from hind limb of WT mice were treated for 1 h with picropodophyllin (PPP; 1 µM), an inhibitor of IGFR-1, prior to the incubation with Ang-(1-7) (10 nM) for 30 min (E) or 24 h (H). Western blot for total and phosphorylated forms of IGFR-1 and Akt were performed. Molecular mass markers are shown in kilodaltons. Quantification of phospho-IGFR-1 (F,I) and phospho-Akt (G,J) normalized to total protein are expressed relative to the vehicle-treated control group and correspond to the mean±s.d. from two independent experiments in triplicate for each experimental condition. *P<0.05 vs vehicle-treated control; ^#^*P*<0.05 vs Ang-(1-7)-treated control.

Finally, the effects of Ang-(1-7) on IGF-1 and IGFR-1 expression, as well as on IGFR-1 phosphorylation, were determined. Vehicle-treated WT/immobilized TA showed decreased IGF-1 expression (Fig. S5A), which was prevented in Ang-(1-7)-treated WT/immobilized TA. In Mas KO/immobilized TA, Ang-(1-7) did not prevent decreased *IGF-1* gene expression (Fig. S5B). In turn, IGFR-1 receptor expression was unchanged in the TA for all conditions in WT and Mas KO mice (Fig. S5C,D). Then, we evaluated the effect of Ang-(1-7) on IGFR-1 phosphorylation. The basal levels of IGFR-1 phosphorylation decreased in vehicle-treated WT TA and Mas KO TA after 1 day of immobilization (Fig. 5A,B), and this effect was still observed after 14 days of immobilization (Fig. 5C). Also, at day 14, Ang-(1-7) prevented decreased IGFR-1 phosphorylation in WT/immobilized TA, but not in the Mas KO/immobilized TA (Fig. 5C,D). Together, these results suggest that the receptor Mas is involved in the recovery of Ang-(1-7)-induced *IGF-1* expression and IGFR-1 phosphorylation at day 14 of immobilization.

Primary culture myotubes were used to evaluate the correlation between Ang-(1-7)-induced activation/phosphorylation of IGFR-1 and Akt. In response to Ang-(1-7) treatment, IGFR-1 was phosphorylated by 24 h post-treatment, whereas Akt showed biphasic kinetics of activation, with early (30 min) and late (24 h) phosphorylation (Fig. S6A,B). To evaluate whether Ang-(1-7)-induced Akt phosphorylation was dependent on IGFR-1 activation, primary myotube cultures were treated with picropodophyllin (PPP), an inhibitor of IGFR-1 activation. Pre-treatment with PPP did not change the Ang-(1-7)-dependent Akt phosphorylation at 30 min (Fig. 5E-G), suggesting an effect independent of IGFR-1 activation. However, PPP pre-treatment decreased the Ang-(1-7)-induced Akt phosphorylation at 24 h concomitant with the inhibition of IGFR-1 phosphorylation (Fig. 5H-J).

These results indicate that Ang-(1-7), together with the Mas receptor, can activate the IGF-1/IGFR-1/Akt pathway and thereby promote IGF-1 expression, in addition to IGFR-1 and Akt phosphorylation. These activities concomitantly inhibited the activity of FoxO3 and activated the phosphorylation of p70S6K.

## DISCUSSION

This is the first report on the anti-atrophic effects of Ang-(1-7) via Mas in disuse skeletal muscle atrophy. Ang-(1-7) treatment maintained muscle strength and prevented decreases in muscle diameter and mass in the immobilized hind limb. Additionally, Ang-(1-7), through Mas, prevented decreased MHC levels while decreasing the expression of atrogin-1 and MuRF-1, two UPP components typically upregulated by disuse. Moreover, the Ang-(1-7)/Mas axis increased the expression of the IGF-1/IGFR-1/Akt pathway, as demonstrated by increased IGF-1 expression; the induction of Akt phosphorylation via IGFR-1; and the activation of p70S6K and FoxO3, two downstream targets of Akt. These Ang-(1-7)-related effects were not observed in Mas KO mice, indicating Mas receptor participation in the anti-atrophic actions of Ang-(1-7).

### RAS axis and disuse muscle atrophy

In the contexts of skeletal muscle physiopathology, the functions of RAS are more thoroughly documented than those of Mas. In classical RAS, Ang II is the main effector molecule, with a role in triggering cachexia muscle wasting (Cabello-Verrugio et al., 2012a, 2015). Our group recently demonstrated that Ang-(1-7), the main peptide of non-classical RAS, has an opposing role to Ang II in cachexia (Cisternas et al., 2015; Meneses et al., 2015). In cases of disuse atrophy, increased Ang II levels have not been reported. However, the pharmacological inhibition of the Ang II receptor, AT1, with losartan decreases disuse skeletal muscle atrophy in older mice (Burks et al., 2011). Interestingly, when AT1 is blocked by losartan, circulating Ang-(1-7) levels increase (Schindler et al., 2007). This indicates that Ang-(1-7) could be mediating the beneficial effects observed for losartan use. Further experiments that evaluate the effects of losartan in Mas KO mice suffering disuse skeletal muscle atrophy would be a relevant step towards elucidating this point.

### Anti-atrophic effect of Ang-(1-7) is dependent on Mas in disuse muscle atrophy

The present data clearly indicate the participation of the Mas receptor in the anti-atrophic effects of Ang-(1-7). This is in agreement with previous reports by our group that used the Mas antagonist A779 (Cisternas et al., 2015; Meneses et al., 2015). Although the participation of Mas in mediating the effects of Ang-(1-7) is well documented, the intracellular pathways directly downstream of the Mas receptor remain unclear. Mas is a G-protein-coupled receptor, the effects of which are mediated by Gq or Gi/o (Solinski et al., 2014). One of the important intracellular G-protein adaptors is β-arrestin, which could be relevant in skeletal muscle atrophy because it has been linked to MAPK and Akt activation. Therefore, a relevant pending issue for further research is to understand the molecular link between Mas and the Akt signalling pathway during skeletal muscle atrophy.

The current study also indicates that Ang-(1-7) could have therapeutic potential in combating disuse atrophy. Resistance exercises that promote adaptive functional and structural responses are widely applied in the treatment of disuse atrophy (Nicastro et al., 2011). Notably, exercise training increases Ang-(1-7) levels and Mas receptor expression in the muscles of rats suffering chronic heart failure (Gomes-Santos et al., 2014). Chronic Ang-(1-7) administration in spontaneously hypertensive rats produces beneficial cardiovascular effects similar to those of exercise training (Bertagnolli et al., 2014). In skeletal muscle, exercise training causes a shift in RAS towards the Ang-(1-7)/Mas axis, which might indicate that the beneficial effects of exercise can be measured by increased Ang-(1-7) levels.

### Role of the Akt pathway in the anti-atrophic effect of Ang-(1-7)

Our data strongly indicate that Akt is a key protein for determining the effects of Ang-(1-7) on skeletal muscle. It is therefore important that Ang-(1-7)-dependent Akt phosphorylation was found to be mediated by the Mas receptor, as inferred through a lack of phosphorylation dependent on Ang-(1-7) in Mas KO mice. The observed Akt activation in the skeletal muscle of Ang-(1-7)-treated mice is in line with previous reports (Cisternas et al., 2015; Munoz et al., 2010). The central role of Akt in skeletal muscle atrophy is supported by studies showing greater muscle atrophy in Akt1- and Akt2-KO mice (Peng et al., 2003). Furthermore, the levels of phosphorylated Akt markedly decrease during unloading-induced muscle atrophy, and events downstream of Akt, such as p70S6K phosphorylation and protein synthesis, also decrease (Hornberger et al., 2001). In regards to this, our group previously showed that Ang-(1-7) reduces Ang-II-mediated atrophic effects in skeletal muscle by restoring muscle strength and mass (Cisternas et al., 2015; Meneses et al., 2015) through a mechanism dependent on Akt phosphorylation (Cisternas et al., 2015). According to the present and previously reported results, Ang-(1-7) might not only activate Akt but could also modulate the main pathway involved in protein synthesis, thereby contributing to the prevention of disuse skeletal muscle atrophy. Further studies are required to evaluate the effect of Ang-(1-7) on protein synthesis.

In addition to favouring protein synthesis, Akt also inhibited protein degradation through the phosphorylation of FoxO transcription factors. When FoxO is phosphorylated, it is maintained in the cytoplasm and prevents the upregulation of the ubiquitin E3 ligases atrogin-1 and MuRF1. By preventing ubiquitination, the subsequent proteasome-induced degradation of myofibrillar proteins, such as MHC, is also impeded (Stitt et al., 2004). During disuse, the IGF-1/Akt pathway is depressed (Childs et al., 2003; Latres et al., 2005), blunting FoxO phosphorylation, consequently leading to increased atrogin-1 and MuRF-1 levels that finally undergo nuclear translation and induce the transcription of the atrogin-1 and *MuRF-1* genes (Sandri, 2008). This study was the first to find Ang-(1-7) stimulating FoxO3A phosphorylation in both basal and disuse conditions. This could indicate that the presence of Ang-(1-7) inhibits the transcriptional activity of FoxO3, thus decreasing the expressions of atrogin-1 and MuRF-1 and the activation of UPP. In turn, this could explain the Ang-(1-7)-mediated inhibition of decreased MHC protein levels. Future studies should consider evaluating the effects of Ang-(1-7) on other pathways involved in protein degradation during muscle atrophy, such as those dependent on calpains and/or autophagy.

### Participation of IGF-1/IGFR-1 pathway on the effect of Ang-(1-7) in disuse muscle atrophy

As mentioned, IGF-1/Akt pathway activity decreases in disuse muscle atrophy (Childs et al., 2003; Latres et al., 2005). The downregulation of IGF-1 signalling in skeletal muscle is related to muscle wasting (Latres et al., 2005), and a transgenic expression of IGF-1 in skeletal muscle has been linked to myofiber hypertrophy (Coleman et al., 1995). In contrast, IGF-1 overexpression reverses muscle wasting in several different models of muscle atrophy (Schakman et al., 2005; Yoshida et al., 2010). Despite these studies, the role of IGF-1 in disuse atrophy is controversial, with some studies suggesting that IGF-1 overexpression does not impact muscle atrophy attenuation (Criswell et al., 1998). The present study found that Ang-(1-7) increased IGF-1 levels and IGFR-1 activation, which could explain the increased Akt phosphorylation and subsequent activation of downstream targets. Supporting these results, a recent study reported that losartan attenuates disuse muscle atrophy by activating the IGF-1/Akt pathway (Burks et al., 2011).

This study is the first to present evidence for the anti-atrophic effects of Ang-(1-7) in disuse muscle atrophy together with the participation of the IGF-1/Akt signalling pathway. Preventing muscle wasting is of major medical importance and is a crucial issue in terms of healthcare costs. Thus, there is an ever-growing need to find new rational therapeutic strategies for the prevention and/or reversal of skeletal muscle atrophy. Such strategies could markedly accelerate rehabilitation following injury or orthopaedic cast immobilization, thereby reducing overall health care costs.

## MATERIALS AND METHODS

### Animals

Twelve-week-old male wild-type (WT) and Mas knockout (Mas KO) (Walther et al., 1998) C57BL/6J mice were used. A lower hind limb was unilaterally immobilized for different time periods using the 3M™ Scotchcast™ Soft Cast Casting Tape (Morales et al., 2015). The mice were randomly separated into four different experimental groups, and three independent experiments were performed. The experimental groups were vehicle-treated WT (PBS), Ang-(1-7)-treated WT, vehicle-treated Mas KO (PBS), and Ang-(1-7)-treated Mas KO. Mice were osmotically infused with Ang-(1-7) (100 ng/kg body weight/min) 24 h before cast immobilization through micropumps (ALZET^®^, Durect, USA), as previously described (Acuna et al., 2014), and maintained during the immobilization period. At the end of each experiment, the animals were euthanized with anaesthesia overdose, and the tibialis anterior (TA) and gastrocnemius (GA) muscles were dissected, removed, rapidly frozen, and stored at −80°C until processing. All protocols were conducted in strict accordance to and with the formal guidance and approval of the Animal Ethics Committee at the Universidad Andrés Bello.

### Muscle histology and muscle fiber determination and quantification

Cryosections (7 µm) of the TA and GA were stained with haematoxylin and eosin (H&E) or Alexa-Fluor^®^-594-tagged WGA (Life Technologies™, USA) according to standard procedures. Fiber sizes were determined by WGA staining and the ImageJ software (NIH, USA), as previously described (Cisternas et al., 2015; Meneses et al., 2015).

### Contractile properties

After immobilization treatments, mice were given an anaesthetic overdose, the TA and GA muscles were removed, and the contractile properties of the muscles were measured as previously described. First, the maximum isometric tetanic force was determined. Following this, the muscles were removed from the bath, and the tendons and any non-muscle tissue were trimmed, blotted once on filter paper, and weighed. Muscle mass and optimum muscle length were used to calculate specific net force, or force normalized to the total muscle fiber cross-sectional area (mN/mm^2^) (Cabello-Verrugio et al., 2012b; Morales et al., 2011, 2013).

### Primary cultures

Adult primary myoblasts were obtained from hind limb muscles of 10- to 20-week-old C57BL/6J mice. Briefly, dissected muscle was digested with collagenase type I (Worthington, USA) for approximately 1 h. A single cell suspension was obtained after filtering the samples through a 70 μm mesh (Falcon). Cells were plated on collagen-coated dishes and cultured for the indicated times in F12-C supplemented with 15% horse serum and 1 nM FGF-2, at 37°C and 5% CO_2_ (Olguin and Olwin, 2004; Olguin et al., 2007). Differentiation was induced by incubating the cultures in F12-C supplemented with 2% horse serum. When required, the inhibitor picropodophyllin (PPP) (Tocris Bioscience, USA; 1 µM) was added for 1 h before treatment with Ang-(1-7).

### Quantitative real-time PCR

The mRNA expressions of mouse atrogin-1, *MuRF-1*, *IGF-1*, *IGFR-1* and the 18S housekeeping gene (TaqMan^®^ Assays-on-Demand™; Applied Biosystems^®^, USA) were quantified using the comparative ΔCt method (2^−ΔΔCT^) (Schmittgen et al., 2000; Winer et al., 1999). The procedures for RNA extraction and reverse transcription were performed as previously described by our group (Morales et al., 2012).

### Immunoblot analysis

Proteins from homogenized muscles were subjected to SDS-PAGE, transferred onto polyvinylidene fluoride membranes (Millipore^®^, USA), and probed with mouse anti-MHC (MF-20; Developmental Studies Hybridoma Bank, University of Iowa, USA), mouse anti-tubulin (Santa Cruz Biotechnology^®^, USA), rabbit anti-phospho Ser^473^ Akt, rabbit anti-total Akt, rabbit anti-phospho Thr^389^ p70S6K, rabbit anti-total p70S6K, rabbit anti-phospho Tyr^1316^ IGFR-1, rabbit anti-total IGFR-1 (Cell Signaling Technology^®^ Inc., USA), rabbit anti-phospho Ser^253^ FoxO3, and rabbit anti-total FoxO3 (Bioss Antibodies™, USA). All immunoreactions were visualized through enhanced chemiluminescence.

### Statistical analysis

For statistical analysis, one or two-way analysis of variance (ANOVA) was used with a post hoc Bonferroni multiple-comparison test (Prism^®^, GraphPad Software, USA). Differences were considered statistically significant at *P*<0.05.
